# Supplementary material for: Multiple P450s and Variation in Neuronal Genes Underpins the Response to the Insecticide Imidacloprid in a Population of Drosophila melanogaster
Source: Sci Rep. 2017 Sep 12;7:11338. doi: 10.1038/s41598-017-11092-5 (PMC5596007; doi:10.1038/s41598-017-11092-5)
Supplement: Supplementary file 1 — Supplementary Figures [file 41598_2017_11092_MOESM1_ESM.pdf]

Title:

Multiple P450s and Variation in Neuronal Genes Underpins the Response to the Insecticide Imidacloprid in a Population of *Drosophila melanogaster*

Authors List:

Shane Denecke[1], Roberto Fusetto[1,2], Felipe Martelli[1], Alex Giang[1], Paul Battlay[1], Alexandre Fournier-Level[1], Richard A. O' Hair[2], Philip Batterham[1*]

1: School of BioSciences, Bio21 Molecular Science and Biotechnology Institute, University of Melbourne, Parkville, Victoria 3010, Australia.

2: School of Chemistry, Bio21 Institute of Molecular Science and Biotechnology, and ARC Centre of Excellence for Free Radical Chemistry and Biotechnology, University of Melbourne, Melbourne, Victoria 3010, Australia

* Correspondence to p.batterham@unimelb.edu.au

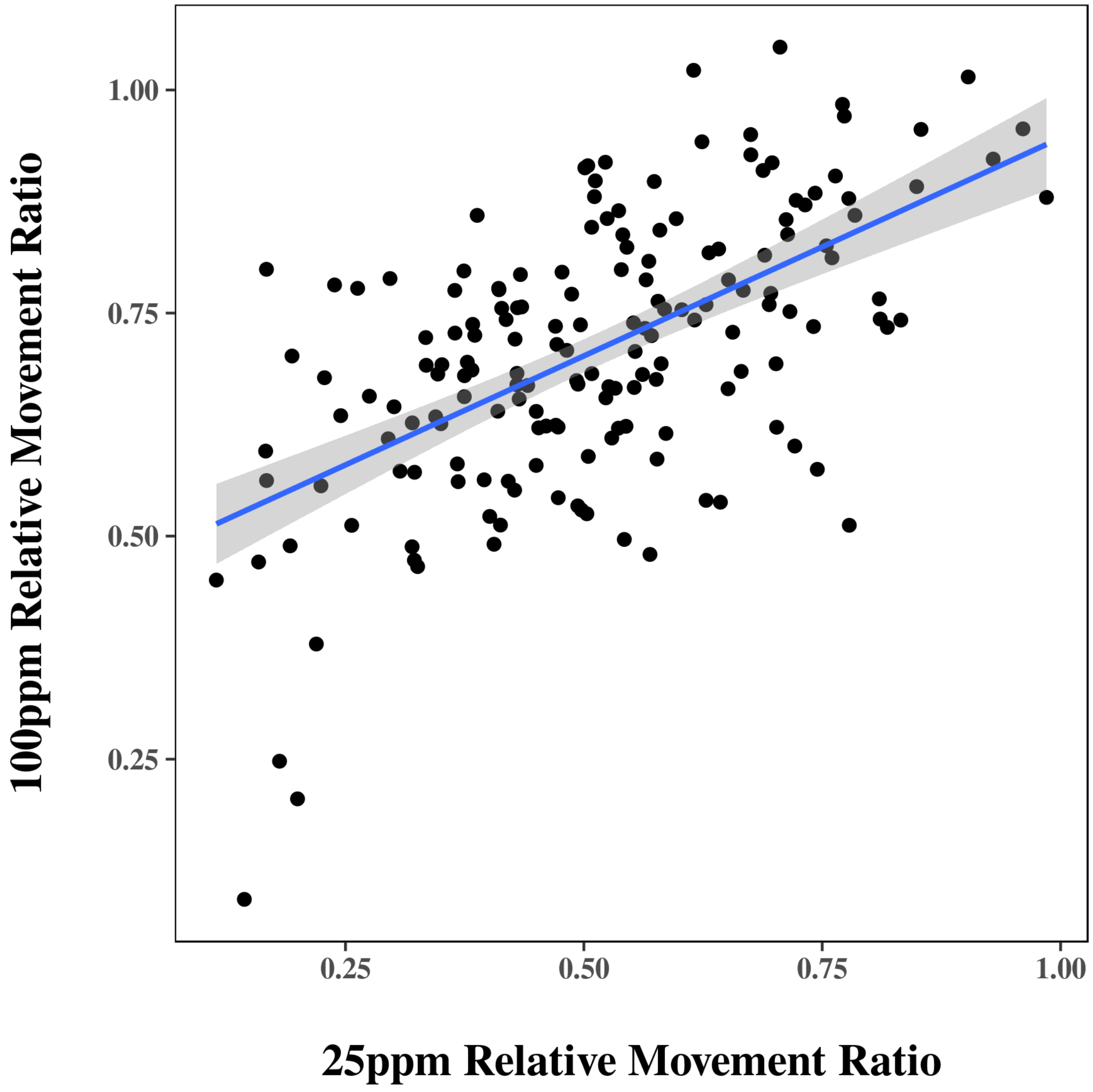

Figure S1- The Correlation of 25 and 100ppm Imidacloprid Response:

For each genotype the 25 and 100ppm mean RMR values were plotted and a linear regression line was fit, showing substantial correlation. The shaded area reflects the 95% confidence interval of the linear model.

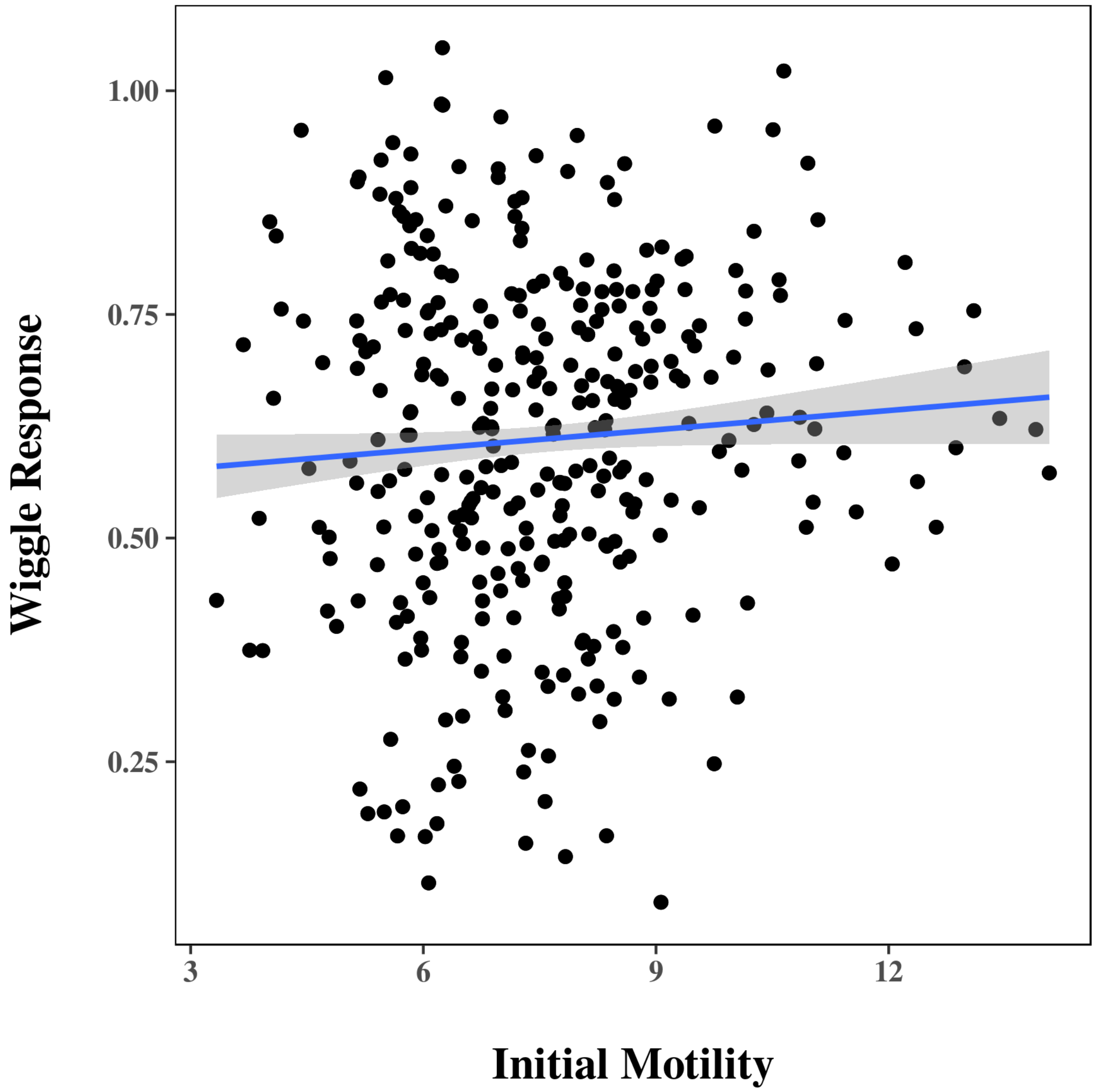

Figure S2- ***The Correlation of Initial Motility with Imidacloprid Response:***

Raw Wiggle Index values at time 0 (x axis) were compared to RMR values after 60 minutes (y axis), suggesting no correlation. The shaded area reflects the 95% confidence interval for the linear model.

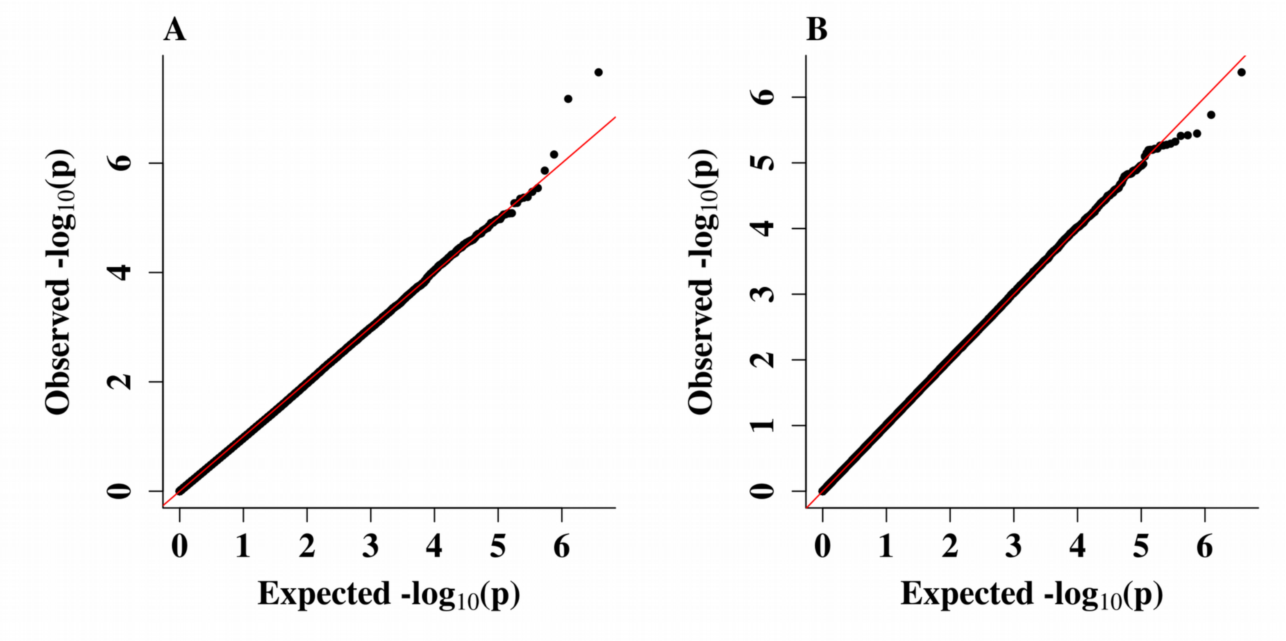

Figure S3- QQ Plots:

Quantile Quantile (QQ) plots were made for the GWAS using A) 25ppm and B) 100ppm RMRs. Each plot shows the expected versus the observed distribution of the significance (p-values) of each annotated variant. The red line indicates where the observed values should fall if they were to match the expected values exactly.

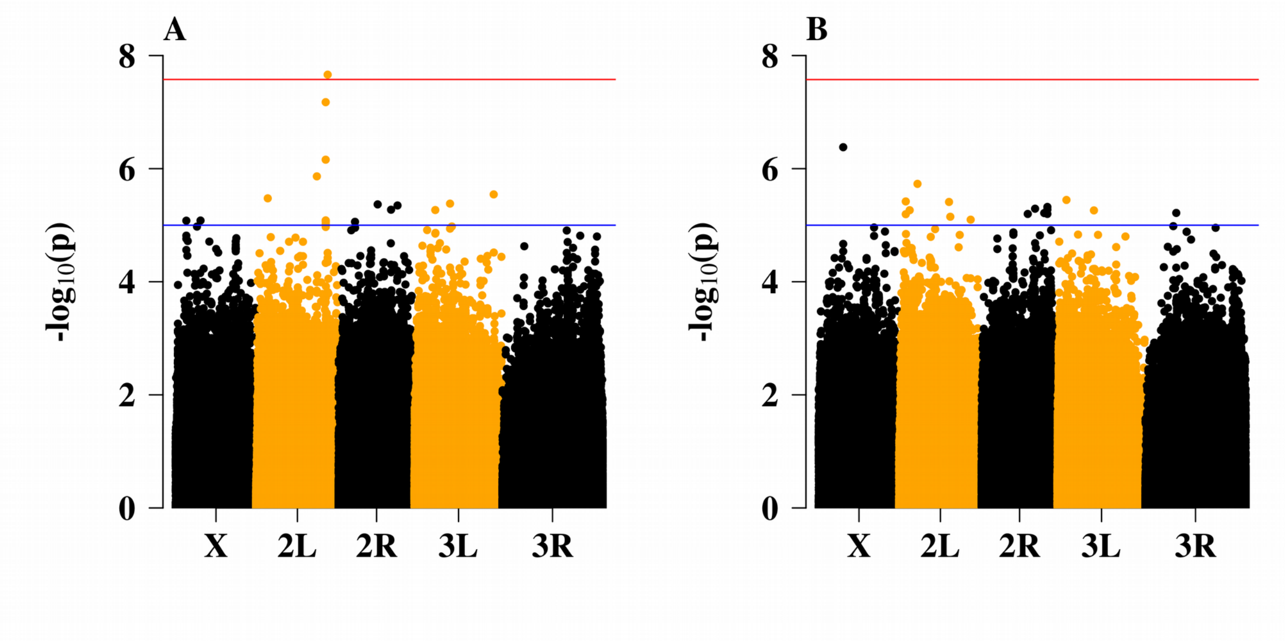

Figure S4- Manhattan Plots:

Manhattan plots were made for the GWAS using A) 25ppm and B) 100ppm RMR values. Each plot shows the significance of a p-value on the Y axis and the position of the genetic variant on the X axis. Genome wide significance thresholds are shown at $p=10^{-5}$ and $p=2.65x10^{-7}$ (Bonferroni threshold)

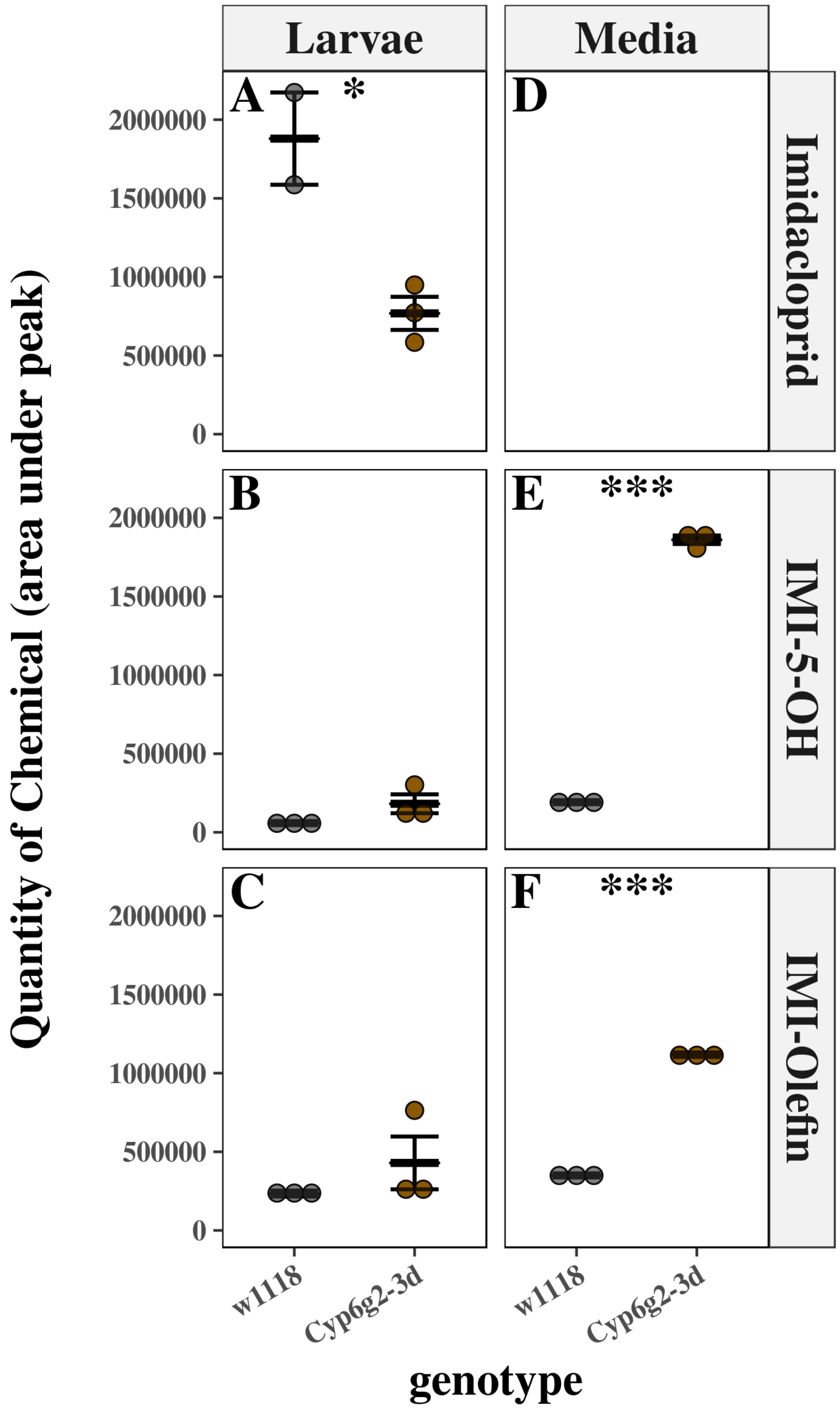

Figure S5- Imidacloprid Metabolism in UAS-Cyp6g2-3d:

The amount of Imidacloprid (A,D) IMI-5-OH (B,E) and IMI-Olefin (C,F) recovered from larval bodies (A-C) or the media (D-F) is reported in HR-GAL4 x w^{1118} (grey) and HR-GAL4 x UAS-Cyp6g2 (brown). No data is presented for imidacloprid in the media, due to the relative abundance of this molecule in the media, which makes detecting changes impossible. Error bars represent standard error of the mean. Stars represent the significance of the difference between the two genotypes measured by he Students T-test corrected for multiple comparisons with the Bonferroni method (=p<.05; **=p<.001; ***=p<.0001).*

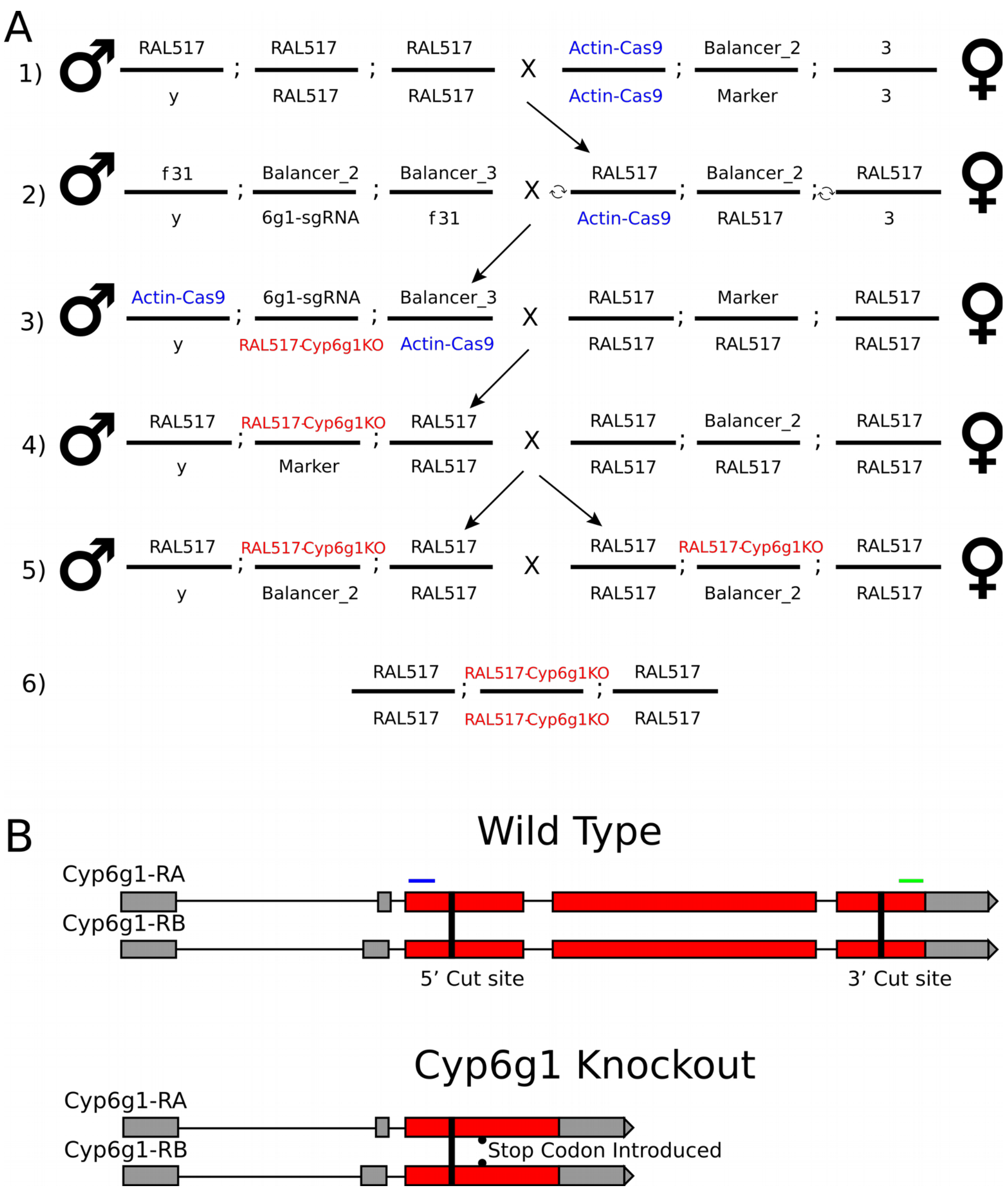

Figure S6- Crossing scheme and Cyp6g1 knock-out:

(A) In step 1, the RAL517 line was crossed to a line that expresses Cas9 under the control of the Actin promoter (Actin-Cas9) with a balanced 2nd chromosome. The Actin-Cas9 cassette is inserted on the X chromosome (highlighted in blue). In step 2, males from the line carrying the balanced Cyp6g1-sgRNA cassette were crossed to females from cross 1. In step 3, backcross males from cross 2 were crossed into a balanced RAL517 background. Because Cas9 and Cyp6g1-sgRNAs are simultaneously expressed in males from cross 2, the deletion of the Cyp6g1 gene will occur at an appreciable frequency. In step 4, males from cross 3 were backcrossed again into a balanced copy of the RAL517 background. This made the X chromosome homozygous for RAL517 and put the deletion (RAL517-Cyp6g1KO) over the CyO balancer. In step 5, males and females from cross 4 carrying RAL517 Cyp6g1 2nd chromosome over the CyO balancer chromosome were crossed together. 6) The homozygous RAL517-Cyp6g1KO flies were identified as the progeny of the cross 5 that appear wild type. indicates recombination. (B) Schematic representation of the Cyp6g1 knock-out outlines that both copies of Cyp6g1 gene are removed from the RAL517 genome. Red boxes indicates coding sequences. The position of the Cyp6g1KO_F and Cyp6g1KO_R primers are reported in blue and green respectively
